# Perceived Effectiveness, Safety, and Attitudes Toward the Use of Nucleic Tests of SARS-CoV-2 Among Clinicians and General Public in China

**DOI:** 10.3389/fpubh.2020.599862

**Published:** 2020-12-17

**Authors:** Ruirui Lan, Robin Sujanto, Kengbo Lu, Zonglin He, Casper J. P. Zhang, Wai-Kit Ming

**Affiliations:** ^1^Department of Public Health and Preventive Medicine, School of Medicine, Jinan University, Guangzhou, China; ^2^International School, Jinan University, Guangzhou, China; ^3^LKS Faculty of Medicine, School of Public Health, The University of Hong Kong, Hong Kong, China

**Keywords:** COVID-19, test kits, questionnaire, Likert scale, nucleic test

## Abstract

**Objective:** To assess whether there is a knowledge gap about the use of test kits for residents and to explore the knowledge, attitudes, and practices of using test kits in China during the coronavirus disease 2019 (COVID-19) epidemic. Method: An online-based, nationwide, and cross-sectional study was conducted. A total of 1,167 respondents were recruited from June 19 to July 2, 2020. All participants completed a validated questionnaire written in Chinese. Electronic consent was obtained from all participants upon their agreement to commence the questionnaire. Perceived efficacy, safety, and their attitudes toward the use of severe acute respiratory syndrome coronavirus 2 (SARS-CoV-2) testing kits were measured.

**Result:** The majority of the study respondents were female [749 (64.2%)], aged 31–40 years old [372 (31.9%)], and located in mainland China [1,137 (97.4%)]. The majority of the respondents held a positive view toward the introduction of the fast-track approval policy for novel coronavirus testing products (6.16 ± 1.30) as well as toward putting more investment in scientific research and biomedicine to improve the detection accuracy of detection kits (5.94 ± 1.55) in China. The respondents valued the detection accuracy more as opposed to the detection time of the testing kits (4.66 ± 2.00), whereas few participants agreed that in the research and development process, detection accuracy could be sacrificed to speed up production and coverage capacity (3.02 ± 2.04).

**Conclusion:** The majority of the participants have a basic knowledge of the detection methods of the SARS-CoV-2 virus and the types of test kits, as well as great confidence in China's domestic production of test kits and decisions. However, how basic knowledge, high compliance, and positive attitudes play a role in easing the tension of the pandemic still remains unknown.

## Introduction

With the fast spread of the severe acute respiratory syndrome coronavirus 2 (SARS-CoV-2), coronavirus disease 2019 (COVID-19) has infected more than 16.6 million people and caused nearly 0.66 million deaths globally, according to the WHO report as of the end of July 2020 (1). Currently, no specific medicine has shown both efficacy and safety in the treatment of COVID-19 (2). Vaccines specifically targeting the viral spike protein or RNA in the market for COVID-19 prevention are still under development (3), while the transmissive ability of SARS-CoV-2 continues to increase with the mutation of the *D614G* gene on the spike protein (4). Therefore, suppression and mitigation strategies, including mask-wearing, social distancing, and quarantining suspected and confirmed cases, are still the major methods to control the spread of the virus (5, 6).

It is essential to distinguish between asymptotic, suspected, or confirmed cases of COVID-19 before quarantine. One who has been exposed to SARS-CoV-2 and has developed symptoms of COVID-19, such as cough, fever, fatigue, etc., is considered as a suspected case and is therefore in need of further identification (7). So far, hundreds of testing kits have been available in the market to meet the exponential demand in testing, targeting antigens, antibodies [immunoglobulin G (IgG) and immunoglobulin M (IgM)], and the viral RNA of SARS-CoV-2 to confirm infection (8, 9). However, antigen tests rarely produce ideal results, and antibody tests generate results that fluctuate in accordance with age, severity, and the time after the manifestation of symptoms. In addition, RNA testing, at times, lacks accuracy as well. For testing kits targeting the RNA of SARS-CoV-2, the sample is often taken from the nose or the throat (10). After undergoing reverse transcription polymerase chain reaction (RT-PCR) in the laboratory, the sample is augmented and cross-matched with the sample to verify the existence of RNA of SARS-CoV-2 (11). However, studies have shown that some COVID-19 patients tested positive again after discharge, and that multiple false-negative RT-PCR-related results were suspected to be related to prolonged nucleic acid transformation time rather than the recurrence of the infection (12). Data from the US show that after testing negative using the RT-PCR, 3.5% of the tested population tested positive in another subsequent RT-PCR test (13). Hence, research suggests that it is optimal to combine the serological total-antibody count and the RT-PCR test to get an enhanced sensitivity of 98.6% and specificity of 98.7% (14).

Since the testing kit is used to identify infected populations, the knowledge, attitude, and practices (KAP) of the residents on testing kits is of utmost importance during the testing process. Current KAP research on COVID-19 is focusing on healthcare workers or the general public in different countries, such as China, the US, and Iran (15–25), or studying personal protective equipment (PPE), namely, face masks, and other non-pharmaceutical interventions (26). In our previous study, we investigated the KAP and compliance with the use of face masks in China and found that most of the respondents showed good basic knowledge on the use of face masks and a good sense of self-protection (Ruirui L et al., Knowledge, Attitude, Practice, and Compliance with the Use of Masks in China against the Current Challenging Pandemic: A Nationwide Cross-sectional Survey, 2020). To our best knowledge, no research has been conducted on the KAP of the general population on testing kits so far. Also, owing to the possible convenient sampling and the fact that the healthcare works or the testing-kits-related occupation may cause false positive or biased results, a nationwide, web-based, cross-sectional survey was conducted in different groups of Chinese residents on COVID-19 diagnosis, knowledge, and confidence on testing kits and personal opinions on specific questions.

## Methods

### Study Design and Population

This is a nationwide, web-based, cross-sectional study. A total of 1,167 respondents were recruited from June 19 to July 2, 2020. All participants completed a validated questionnaire written in Chinese. Electronic consent was obtained from all participants upon their agreement to commence the questionnaire. Perceived efficacy, safety, and their attitudes toward the use of a testing kit of SARS-CoV-2 were measured.

### Study Tool

The survey questionnaire was designed in Chinese and translated to English. Two experts were asked to review the questionnaire in order to make sure that it reflected the knowledge and attitude of the Chinese population on the use of the COVID-19 testing kit. Accordingly, the questionnaire was further modified to meet the aim required. In the questionnaire, single-choice, multiple-choice, and Likert 7-point scales were used. Following an informative consent form, the final closed-ended questionnaire consisted of 22 questions. The questionnaire was divided into three sections: (1) 13 questions for the perceived knowledge of the use of testing kits for SARS-CoV-2, (2) 5 questions for the attitudes toward the use of testing kits for SARS-CoV-2, and (3) 4 questions for the Likert scale of attitudes toward the use of testing kits for SARS-CoV-2. The internal reliability (KR-20) for this questionnaire was 0.80, and the Kaiser–Meyer–Olkin measure of sampling adequacy was 0.632.

### Data Collection

The participants were recruited *via* peer referral in the selected cohorts, and data were collected using an anonymous online questionnaire survey platform powered by *WenJuanXing* (www.wjx.cn). The questionnaires were distributed *via* WeChat, a Chinese cell/web app for messaging, social media, and communications, where a unique two-dimensional code directing to the questionnaire was sent to the potential participants. The data of the questionnaire would be collected only if the entire questionnaire was finished.

### Statistical Analysis

The questionnaire established strata by age group (<20, 20–30, 31–40, 41–50, 51–60, or >60 years), sex (male or female), current location (Mainland China, Hong Kong, Macau, Taiwan, or Overseas), education level (below senior high school, undergraduate, or graduate and above), and the role they played in the process of implementing the SARS-CoV-2 test kit in the general population (R&D personnel, production personnel, sales personnel, healthcare workers who do not operate directly, healthcare workers who operate directly, or ordinary people being tested).

The data obtained from the participants were analyzed using Stata MP 14.0 (Stata Corp., USA). Means with standard deviations were calculated for continuous variables and frequency with percentages for categorical variables. No sampling weights were used. Knowledge scores were compared using an independent sample *t*-test for differences in mean score between two groups of variables, and analysis of variance (ANOVA) was used for comparison between multiple groups. A *P*-value < 0.05 was considered significant.

## Results

### Descriptions of Demographics of the Respondents

A total of 1,167 results were analyzed in the study. The baseline characteristics were shown in Table 1. The majority of the study respondents were female [749 (64.2%)], aged 31–40 years old [372 (31.9%)], and located in Mainland China [1,137 (97.4%)]. Undergraduate respondents contributed most [636 (54.5%)], and the main population of participants being tested was ordinary people [643 (55.1%)].

**Table 1:** Baseline characteristics.

**Baseline characteristics**	**Value**
***N***	**1,167**
**Gender**	
Male	418 (35.8%)
Female	749 (64.2%)
**Age group**	
<20	36 (3.1%)
20–30	344 (29.5%)
31–40	372 (31.9%)
41–50	293 (25.1%)
51–60	101 (8.7%)
>60	21 (1.8%)
**Current location**	
Mainland China	1,137 (97.4%)
Hong Kong, Macau, and Taiwan	17 (1.5%)
Overseas	13 (1.1%)
**Education level**	
Below senior high school	111 (9.5%)
Undergraduate	636 (54.5%)
Graduate and above	420 (36.0%)
**What role do you play in the process of implementing the SARS-CoV-2 test kit in the general population?**	
R&D personnel	15 (1.3%)
Production personnel	7 (0.6%)
Sales personnel	19 (1.6%)
Healthcare workers (do not operate directly)	466 (39.9%)
Healthcare workers (operate directly)	17 (1.5%)
Ordinary people being tested	643 (55.1%)

### The Perceived Knowledge of the Use of a Testing Kit for SARS-CoV-2

**Figure 1:**
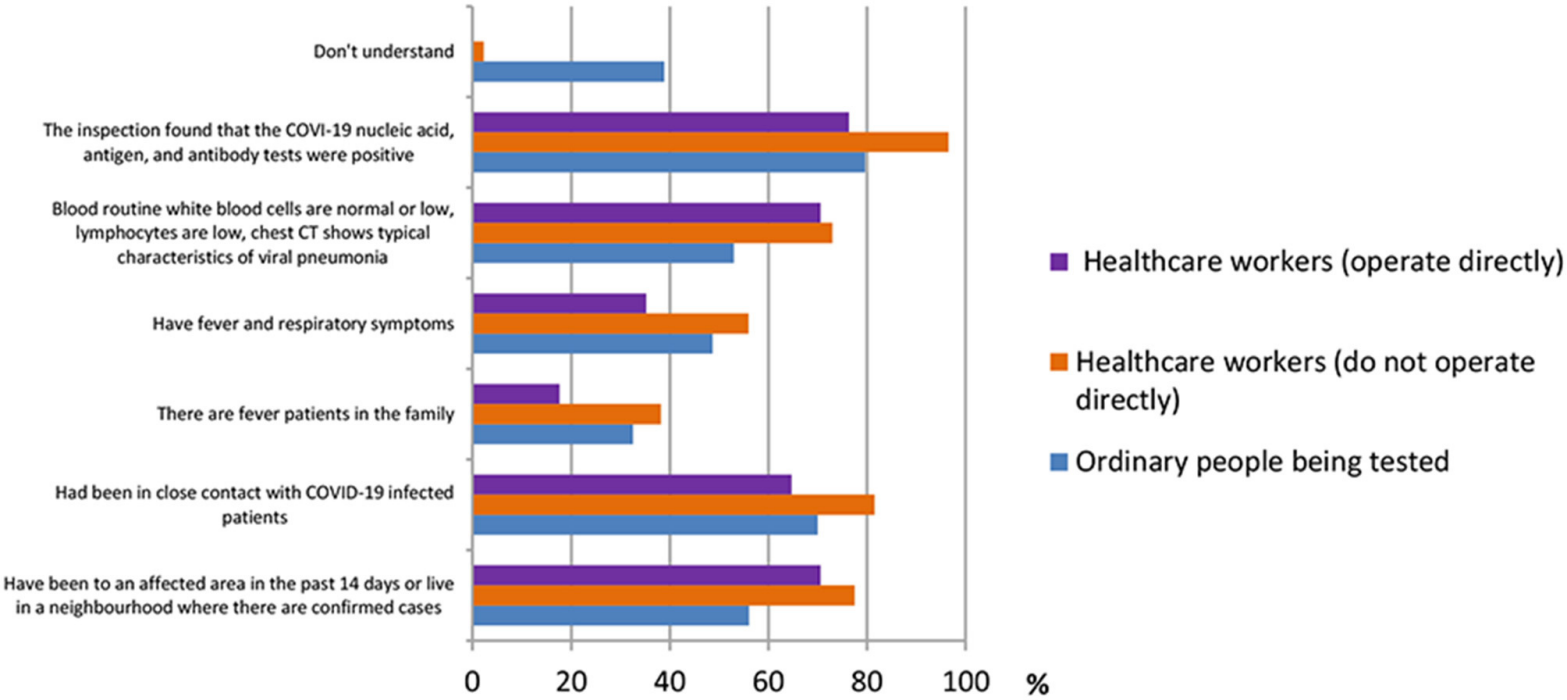
Distribution of responses to the question, “How to identify COVID-19.”

**Figure 2:**
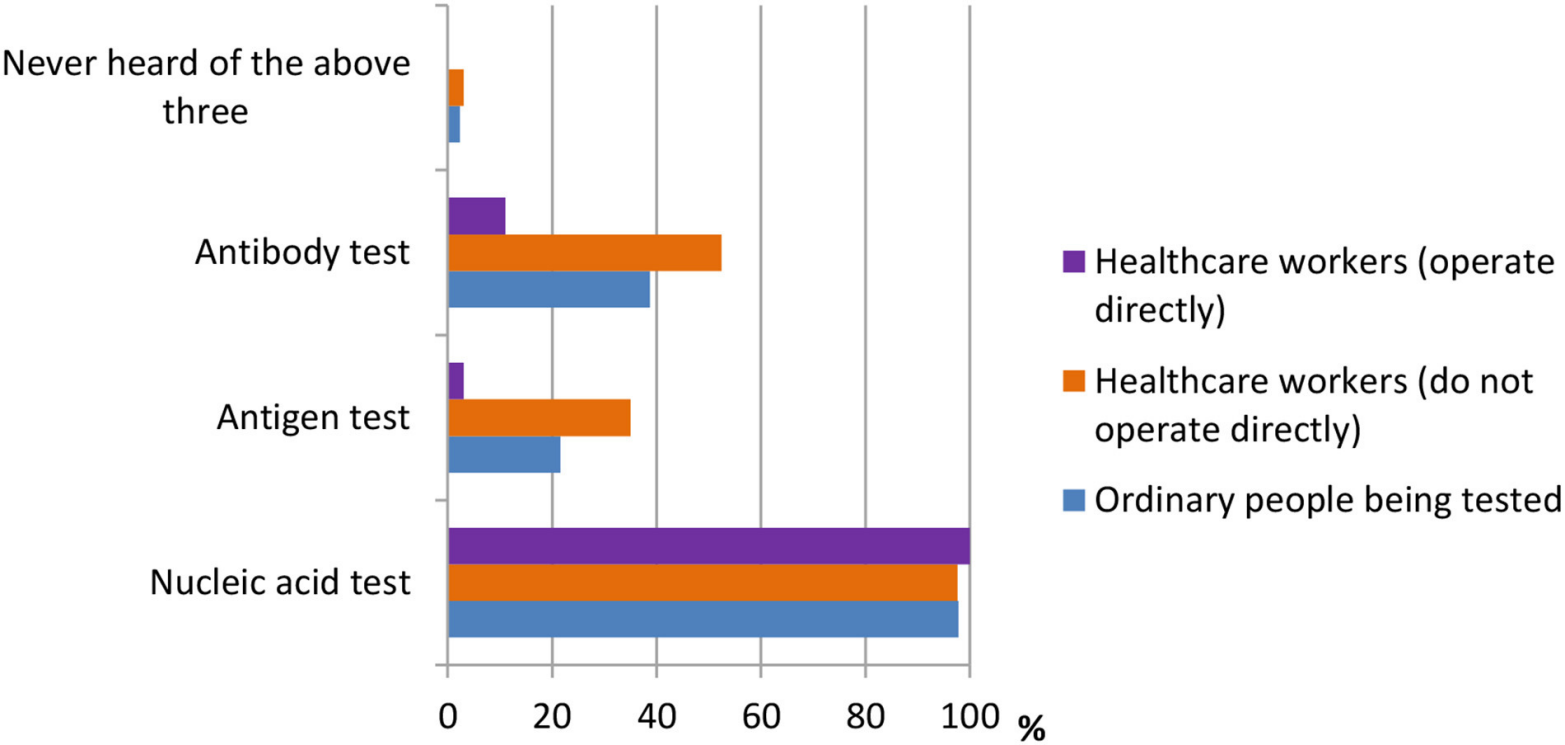
Distribution of responses to the question, “Which means of detection have you heard of.”

A total of 13 questions were designed in this section. The questions were shown in Table 2. According to the results shown in Figure 1, many people had different thoughts about the identification of COVID-19. The result was classified into three groups, namely, ordinary people being tested (OP), healthcare workers who do not operate directly (HW1), and healthcare workers who operate directly (HW2). In the question “How do you identify a suspect of COVID-19?” the majority of the three groups of participants {OP [512 (79.6%)], HW1 [450 (96.6%)}, and HW2 [13 (76.5%)]) believed that “The COVID-19 nucleic acid, antigen, and antibody tests were positive” was the right answer. In the question “Which means of COVID-19 detection have you heard of during this pandemic?” the nucleic acid test was the detection method most heard among three groups {OP [629 (97.8%)], HW1 [455 (97.6%)], and HW2 [17 (100%)]}, whereas the antigen test {HW1 [163 (35.0%)], HW2 [3 (17.6%)]} and the antibody test {HW1 [244 (52.4%)], HW2 [11 (64.7%)]} were more often heard among healthcare workers, according to Figure 2.

**Table 2:** The perceived knowledge of the use of testing kit for SARS-CoV-2.

**Factor**	
***N***	**1,167**
**How do you identify a suspect of COVID-19?**	
(Had been in close contact with COVID-19 infected patients)	863 (74.0%)
(There are fever patients in the family)	403 (34.5%)
(Have fever and respiratory symptoms)	592 (50.7%)
(Blood routine white blood cells are normal or low, lymphocytes are low, chest CT shows typical characteristics of viral pneumonia)	716 (61.4%)
(The inspection found that the COVID-19 nucleic acid, antigen, and antibody tests were positive)	939 (80.5%)
(Do not understand)	40 (3.4%)
**Which means of COVID-19 detection have you heard of during this pandemic?**	
(Nucleic acid test)	1,137 (97.4%)
(Antigen test)	317 (27.2%)
(Antibody test)	523 (44.8%)
(Never heard of the above three)	31 (2.7%)
**Which means of COVID-19 detection have you used during this pandemic?**	
(Nucleic acid test)	758 (65.0%)
(Antigen test)	83 (7.1%)
(Antibody test)	169 (14.5%)
(Never heard of the above three)	410 (35.1%)
**According to your knowledge, how long does it take to complete a coronavirus nucleic acid test?**	
(In 5 min)	44 (3.8%)
(5–30 min)	109 (9.3%)
(30–60 min)	100 (8.6%)
(1–12 h)	350 (30.0%)
(12–24 h)	290 (24.9%)
(More than 24 h)	239 (20.5%)
(Do not understand)	183 (15.7%)
**What is the perceived accuracy rate of the nucleic acid test in China?**	
(Below 30%)	26 (2.2%)
(30–60%)	175 (15.0%)
(60–90%)	349 (29.9%)
(Above 90%)	378 (32.4%)
(Do not understand)	284 (24.3%)
**Which of the following products have you heard of or used?**	
[SARS-CoV-2 nucleic acid detection kit (fluorometric real-time PCR)]	535 (45.8%)
[SARS-CoV-2 nucleic acid detection kit (thermostatic amplitude-real-time fluorescence method)]	173 (14.8%)
[SARS-CoV-2 nucleic acid detection kit (hybrid capture immunofluorescence method)]	112 (9.6%)
[SARS-CoV-2 nucleic acid detection kit (RNA capture probe method)]	130 (11.1%)
[SARS-CoV-2 nucleic acid detection kit (combined probe—anchored polymerization sequencing method) and supporting instruments and software]	97 (8.3%)
[Six respiratory virus nucleic acid detection kits (thermostatic amplification chip method) and supporting instruments]	102 (8.7%)
(Have not heard of any of these)	507 (43.4%)
**How long do you think the nucleic acid test/serology test takes?**	
(in 5 min)	38 (3.3%)
(5–30 min)	114 (9.8%)
(30–60 min)	139 (11.9%)
(1–12 h)	232 (19.9%)
(12–24 h)	172 (14.7%)
(Above 24 h)	167 (14.3%)
(Do not understand)	417 (35.7%)
**From your understanding, what is the current accuracy of antigen/antibody detection?**	
(Below 30%)	29 (2.5%)
(30–60%)	116 (9.9%)
(60–90%)	281 (24.1%)
(Above 90%)	333 (28.5%)
(Do not understand)	457 (39.2%)
**Which antigen/antibody detection tests have you used before?**	
(Colloidal gold products)	230 (19.7%)
(Magnetic particle chemiluminescence products)	89 (7.6%)
(None of them)	897 (76.9%)
**Which means of testing would you prefer, an antigen detection kit, antibody detection kit, or a nucleic acid detection kit?**	
(Antigen detection kit)	66 (5.7%)
(Antibody detection kit)	105 (9.0%)
(Nucleic acid detection kit)	612 (52.4%)
(I am not sure)	384 (32.9%)
**Do you think the testing kit is feasible for screening normal people?**	
(Yes, it is feasible)	854 (73.2%)
(No, it is not feasible)	105 (9.0%)
(I am not sure)	208 (17.8%)
**Personally, do you trust a non-professional operating the testing kits?**	
(Yes, I do)	210 (18.0%)
(No, I do not)	795 (68.1%)
(I am not sure)	162 (13.9%)
**Which sampling method do you think is the most accurate?**	
(Throat swab)	955 (81.8%)
(Nasopharyngeal swab)	661 (56.6%)
(Anal swab)	137 (11.7%)
(Sputum)	323 (27.7%)
(Bronchoalveolar lavage fluid)	324 (27.8%)
(Saliva)	167 (14.3%)
(Blood)	343 (29.4%)
(Urine)	80 (6.9%)
(Do not understand)	92 (7.9%)

**Figure 3:**
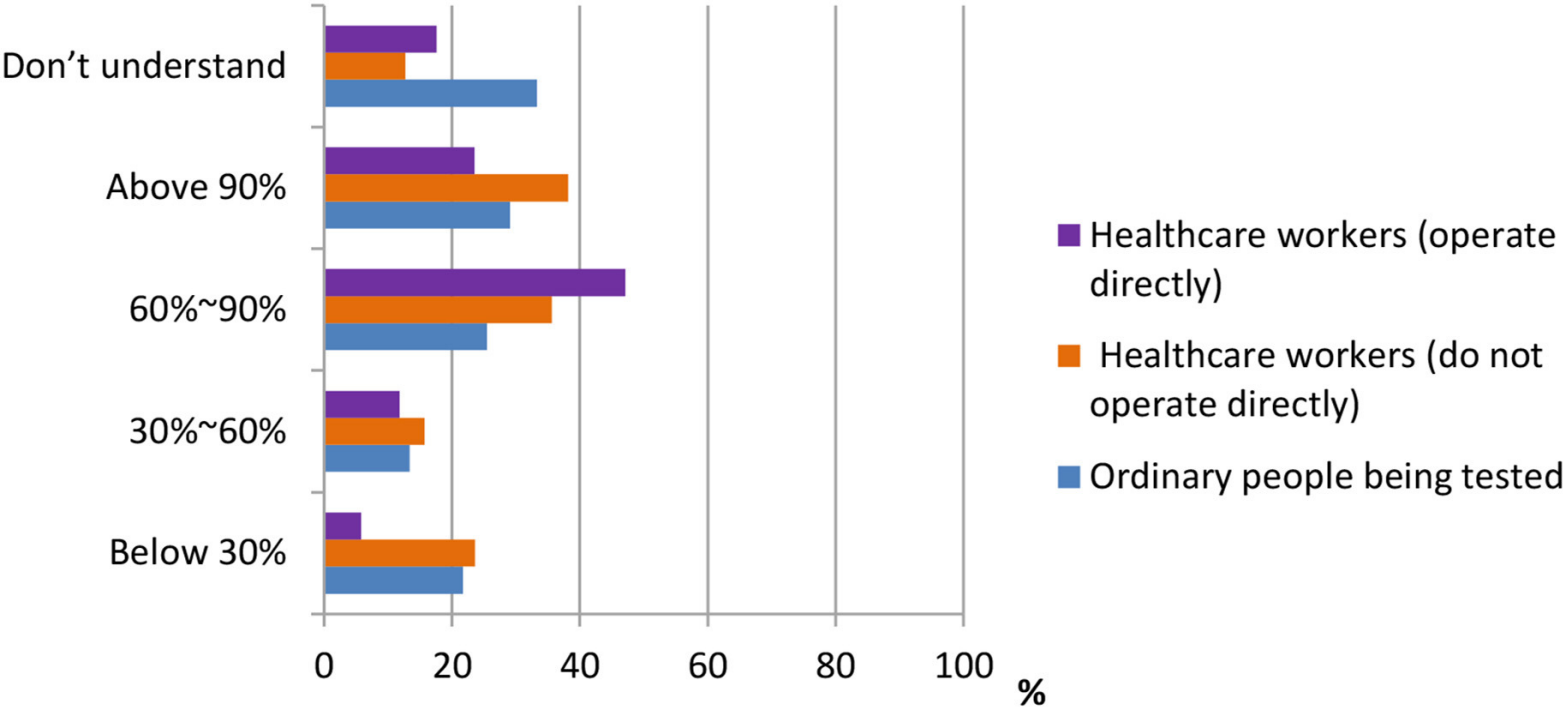
Distribution of responses to the question, “Preceived accuracy rate of nucleic acid test in China.”

**Figure 4:**
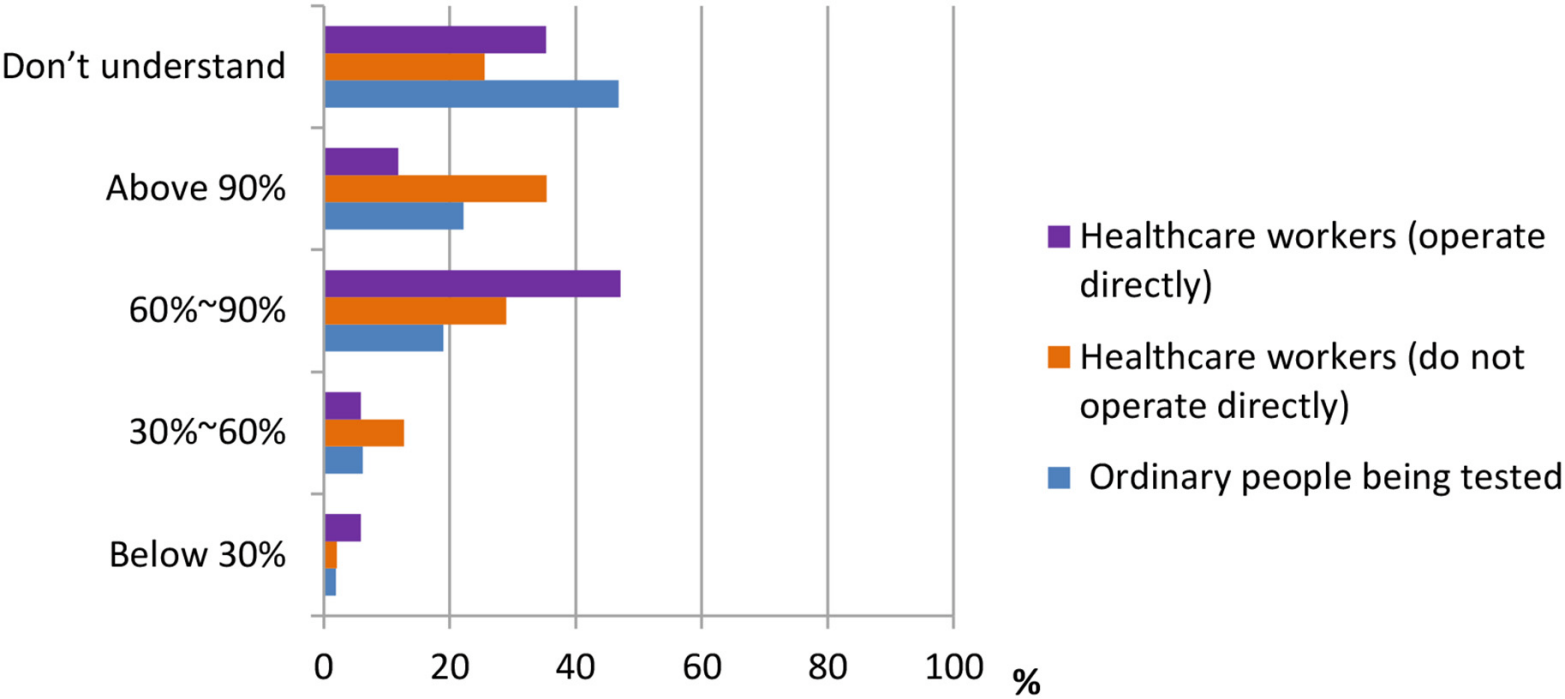
Distribution of responses to the question, “Current accuracy of antigen/antibody detection.”

According to the data shown in Figures 3, 4, three groups of participants had different answers on the accuracy rate of the nucleic acid test, the antigen test, and the antibody test. From the data, we observed that most ordinary people being tested do not understand the accuracy rate of nucleic acid test and antigen/antibody test. Only 33.3 and 46.8% do. On the other hand, more percentage of healthcare workers who do not operate directly [178 (38.2%), 165 (35.4%)] chose “above 90%” in both nucleic acid test and antigen/antibody test, whereas most of the healthcare workers who operate directly chose the answer “60–90%” in both questions.

**Figure 5:**
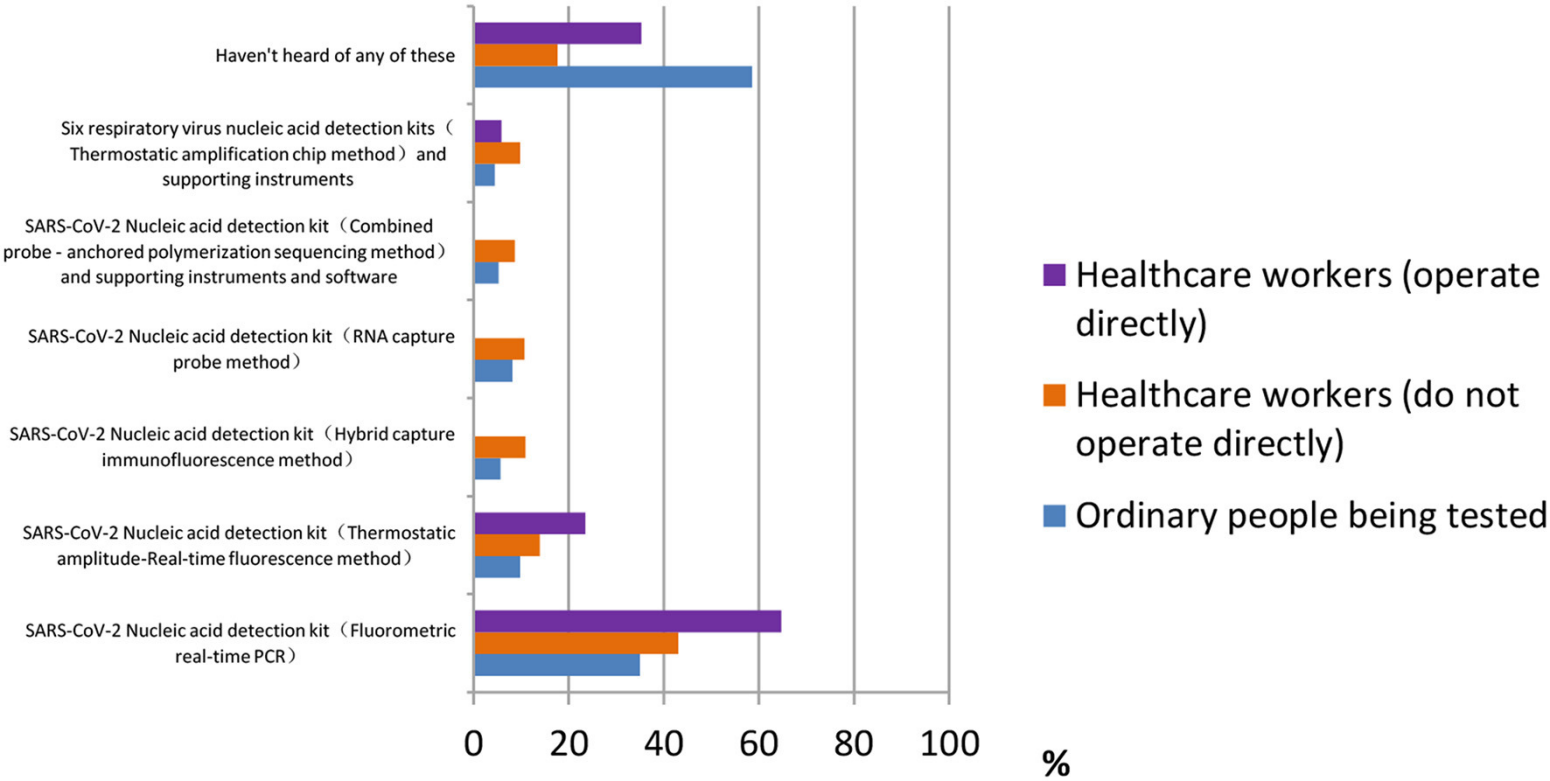
Distribution of responses to the question, “Which of the following products have you heard of or used.”

For the question “Which of the following products have you heard of or used,” most ordinary participants [377 (58.6%)] did not understand any of the products, according to Figure 5. Healthcare workers who do not operate directly were most familiar with the SARS-CoV-2 nucleic acid detection kit (fluorometric real-time PCR) [277 (64.7%)]. The majority of healthcare workers who operate directly also chose this answer [11 (43.1%)]. We learn from the figure that many products are still alien to the public.

**Figure 6:**
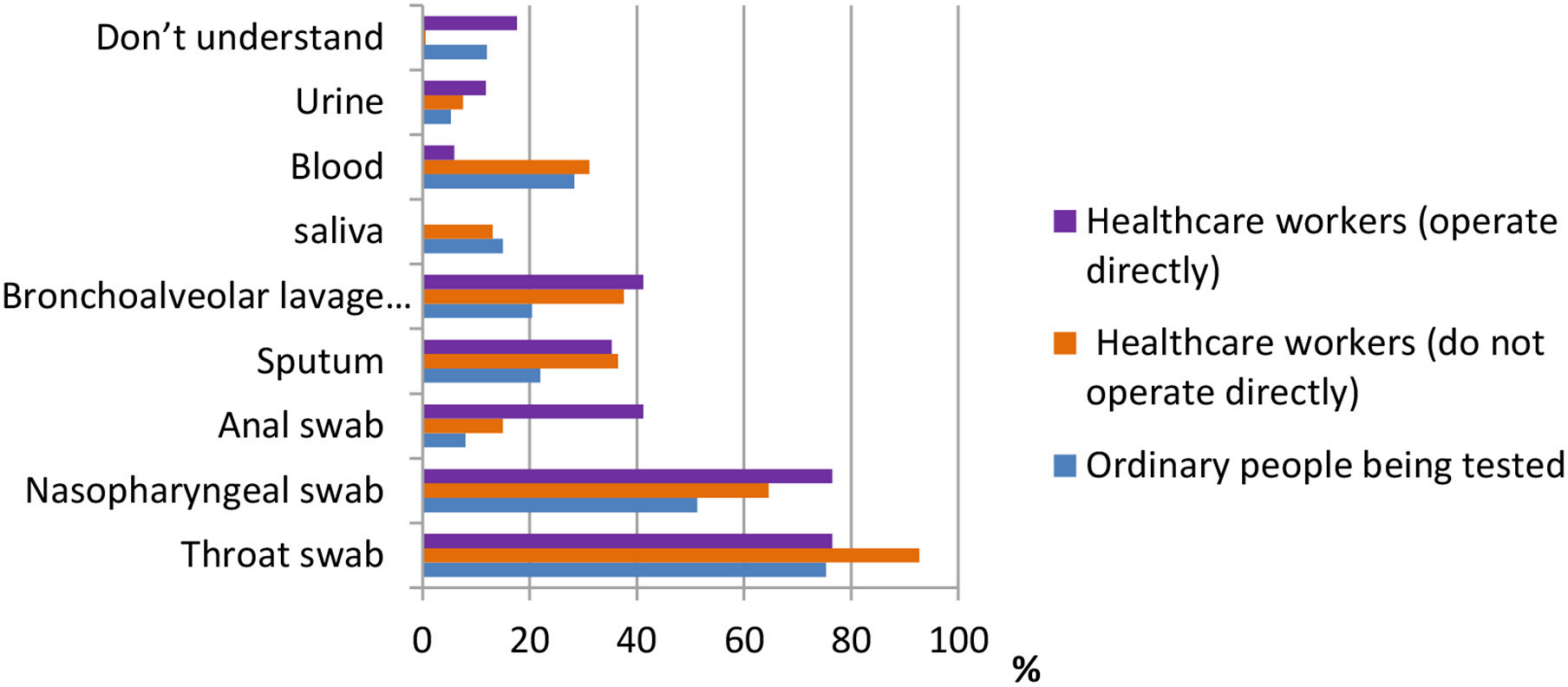
Distribution of responses to the question, “Which sampling method is the most accurate.”

One of the most important pieces of information in terms of knowledge of COVID-19 is the sampling method. According to the data shown in Figure 6, for ordinary participants, the answers throat swab [484 (75.3%)], nasopharyngeal swab [330 (51.3%)], and blood test [182 (28.3%)] were the most common. For healthcare workers who do not operate directly, the answers throat swab [432 (92.7%)], nasopharyngeal swab [301 (64.6%)], and bronchoalveolar lavage fluid [175 (37.6%)] were the most common. Finally, for healthcare workers who operate directly, the answers throat swab [13 (76.5%)], nasopharyngeal swab [13 (76.5%)], and bronchoalveolar lavage fluid and anal swab [both 7 (41.2%)] were the most common.

### The Attitudes Toward the Use of Testing Kits for SARS-CoV-2

**Figure 7:**
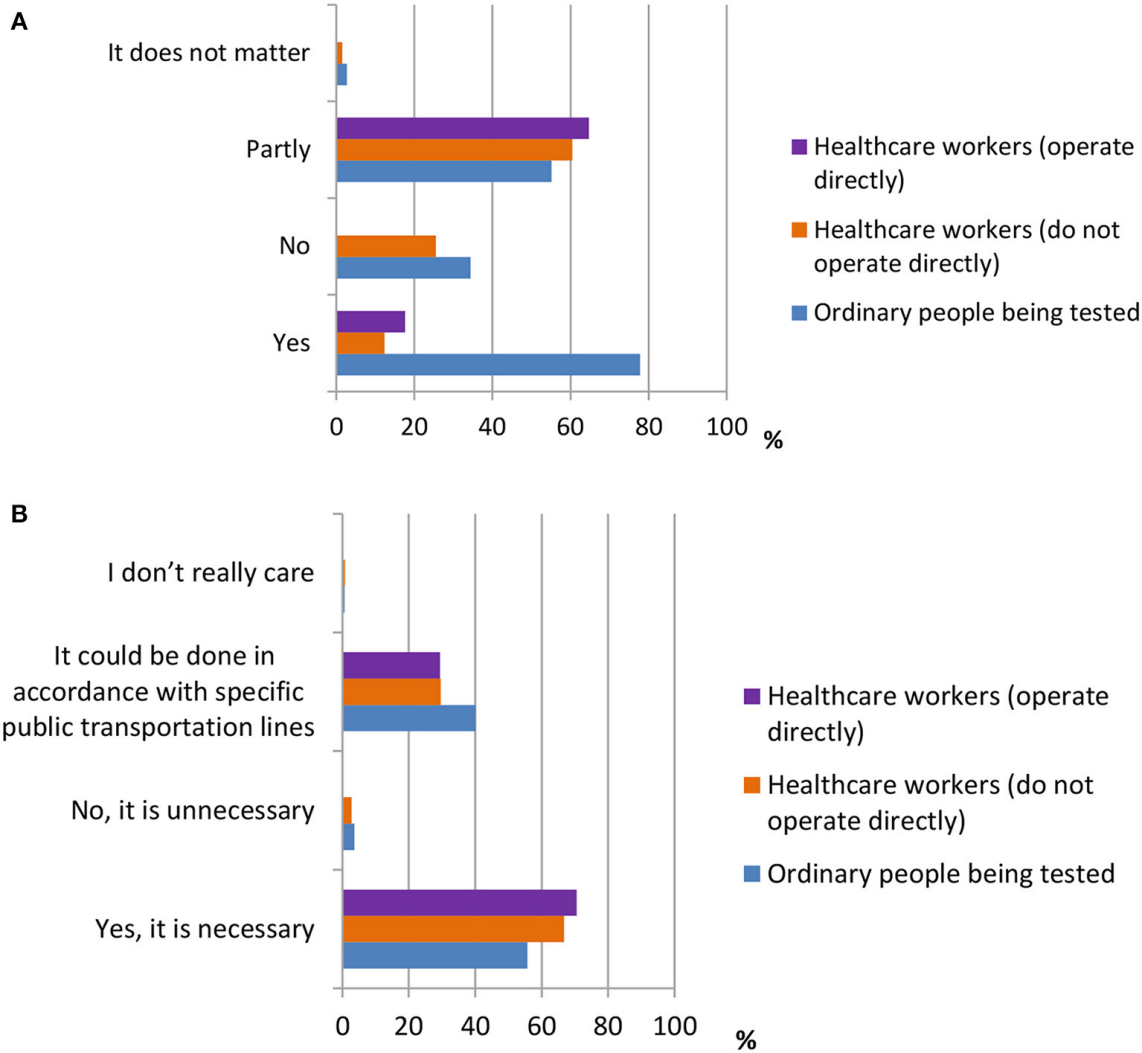
The attitudes towards the use of testing kit for SARS-CoV-2. **(A)** Distribution of responses to the question, “Do you think the residents being tested should self-pay the testing?” **(B)** Distribution of responses to the question, “Do you think it is necessary to set up detection centers at places where there is high population density.”

Five questions were designed to investigate attitudes toward the use of SARS-CoV-2 testing kits. The questions are shown in Table 3. According to the data shown in Figure 7, the majority of ordinary participants [354 (77.8%)] agreed that residents being tested should self-pay for the testing, whereas the majority of healthcare workers [282 (60.5%), 11 (64.7%)] thought that they should only be required to pay part of the testing fee. In addition, the majority of all three groups of participants {OP [358 (55.7%)], HW1 [311 (66.7%)], and HW2 [12 (70.5%)]} agreed that it is necessary to set up detection centers at places with high population densities, such as airports, bus stations, and ports.

**Table 3:** The attitudes toward the use of testing kit for SARS-CoV-2.

**Factor**	**Level**
***N***	**1,167**
**Testing kits are produced both domestically and abroad. Which kind of testing kit do you prefer?**	
Those produced domestically	822 (70.4%)
Those produced abroad	81 (6.9%)
I do not care	264 (22.6%)
**Do you think the residents being tested should self-pay the testing?**	
Yes	115 (9.9%)
No	353 (30.2%)
Partly	667 (57.2%)
It does not matter	32 (2.7%)
**Do you think it is necessary to set up detection centers at places where there is high population density, such as airports, bus stations, and ports?**	
Yes, it is necessary	706 (60.5%)
No, it is unnecessary	37 (3.2%)
It could be done in accordance with specific public transportation lines	414 (35.5%)
I do not really care	10 (0.9%)
**Which one do you think may contribute to the false-positive result?**	
(Cross contamination of instruments or reagents)	680 (58.3%)
(Misoperation)	548 (47.0%)
(The person being tested is at a specific stage of disease development)	712 (61.0%)
(Other reasons)	49 (4.2%)
(Do not understand)	205 (17.6%)
**What do you think contributes to a false-negative result? (check all that apply)**	
The kit's sensitivity is too low	749 (64.2%)
Not enough samples were extracted to produce accurate results	714 (61.2%)
The samples were extracted too soon or too late	477 (40.9%)
The sample was not properly extracted (too high—extracted samples from the oral cavity or too low—extracted samples from the lung)	547 (46.9%)
The tested population used antibiotic drugs prior to the nucleic acid test	372 (31.9%)
Instrumental error	422 (36.2%)
Improper operation	494 (42.3%)
Other reasons	18 (1.5%)
I do not know	181 (15.5%)

**Figure 8:**
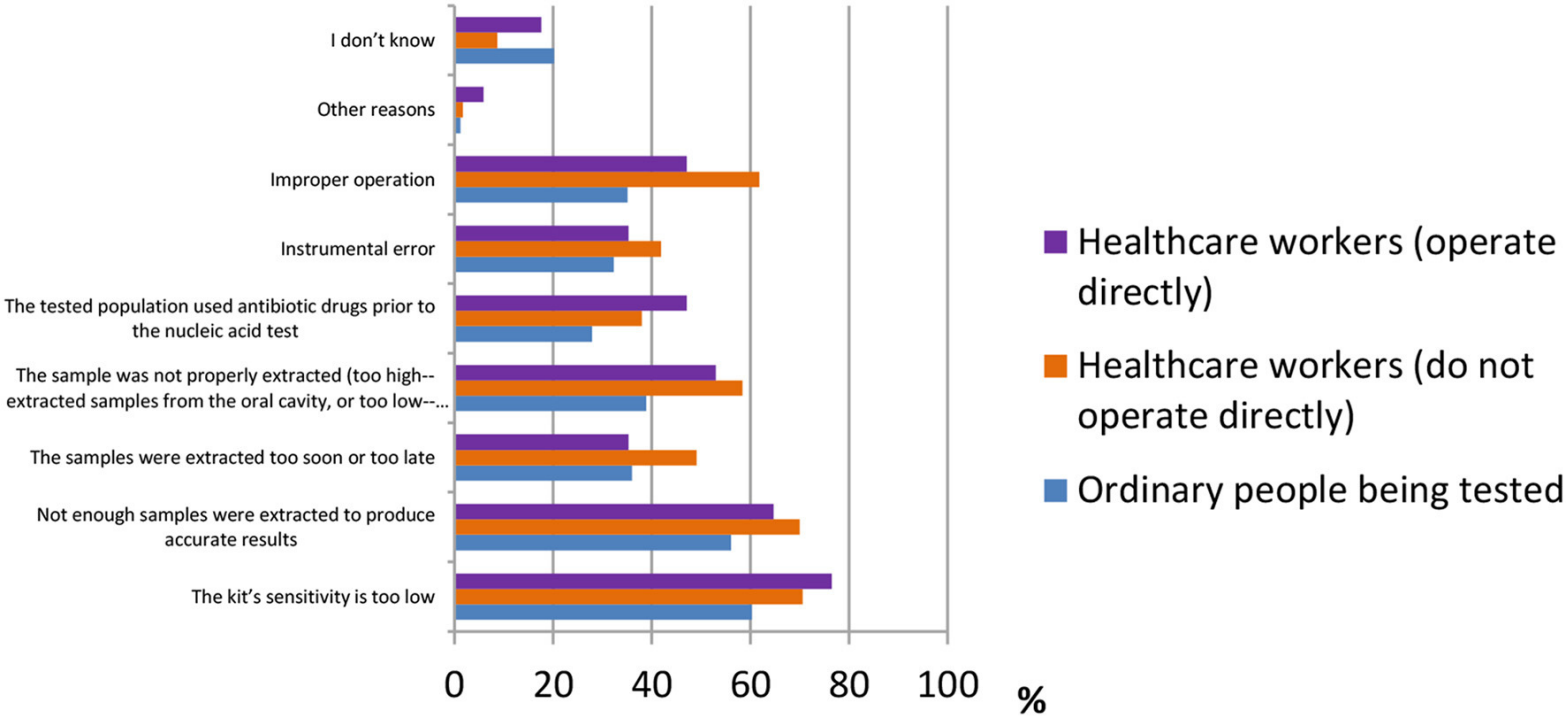
Distribution of responses to the question, “Causes of false-negative results.”

False-negative results of COVID-19 detection are still a serious issue all over the world. Many factors may cause a false-negative result. According to the results shown in Figure 8, the majority of ordinary people believed “the kit's sensitivity is too low” [388 (60.3%)], followed by “not enough samples were extracted to produce accurate results” [361 (56.1%)] and “the sample was not properly extracted (too high—extracted samples from the oral cavity or too low—extracted samples from the lung)” [250 (38.9%)]. Healthcare workers who operate directly had the same answers as ordinary participants [13 (76.5%), 11 (64.7%), and 9 (53%)]. However, healthcare workers who do not operate directly answered “improper operation” [288 (61.8%)] as the third main reason for causes of false-negative results, whereas the other two were the same as above [329 (70.6%), 326 (70%)].

### Different Age Groups in the Knowledge of Testing Kits for SARS-CoV-2

**Figure 9:**
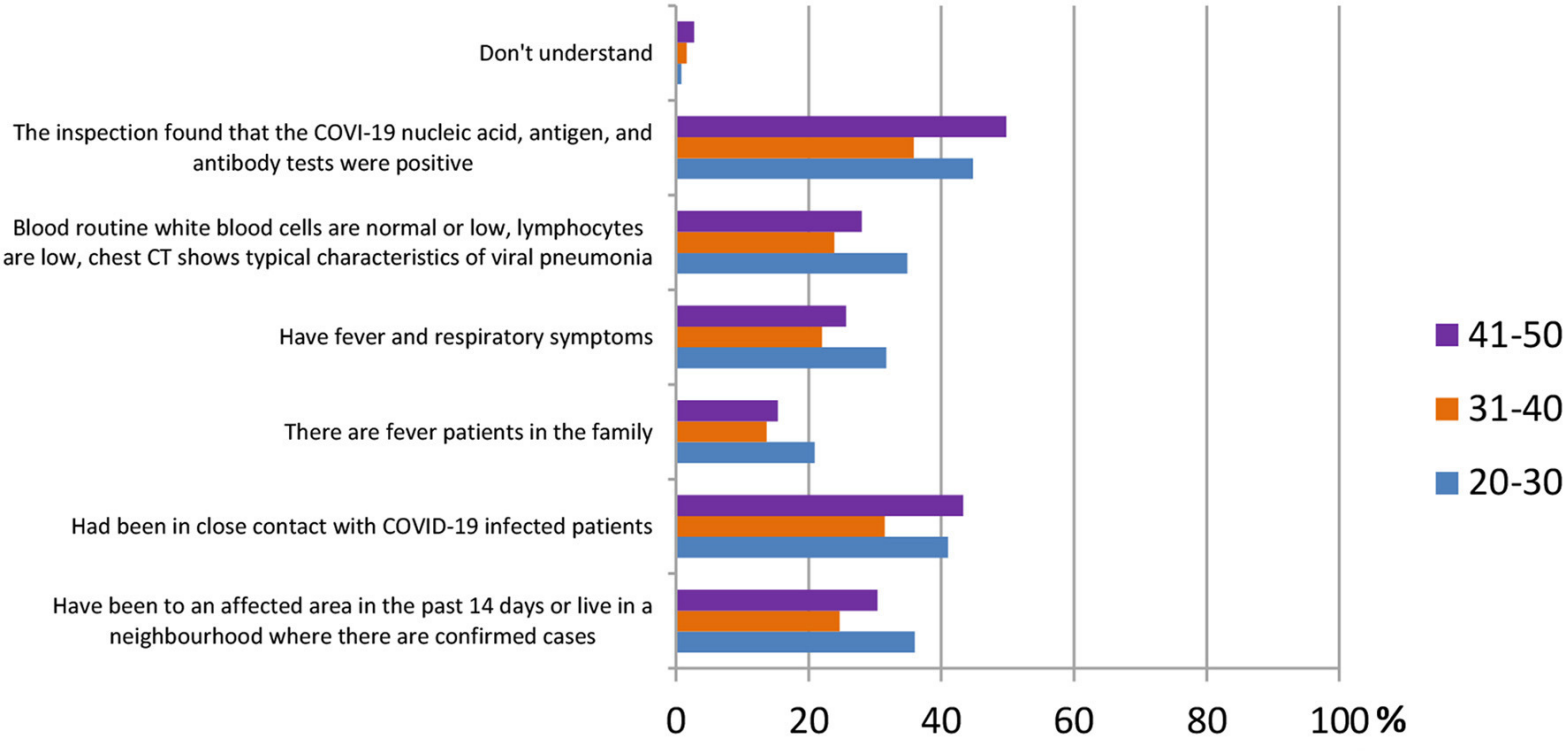
Knowledge of testing kit for SARS-Co V-2 in different age groups.

In this section, ordinary people being tested for COVID-19 were selected to observe whether different age groups had different answers regarding their knowledge of SARS-CoV-2 testing kits. Three main population groups were selected, from ages 20 to 30 (344 participants), 31 to 40 (372 participants), and 41 to 50 (293 participants). According to the results shown in Figure 9, for the question “how to identify a suspect of COVID-19,” the answer “inspection found that the COVID-19 nucleic acid, antigen, and antibody tests were positive” was agreed on by the majority of all age groups [154 (44.8%), 133 (35.8%), and 146 (49.8%)].

**Figure 10:**
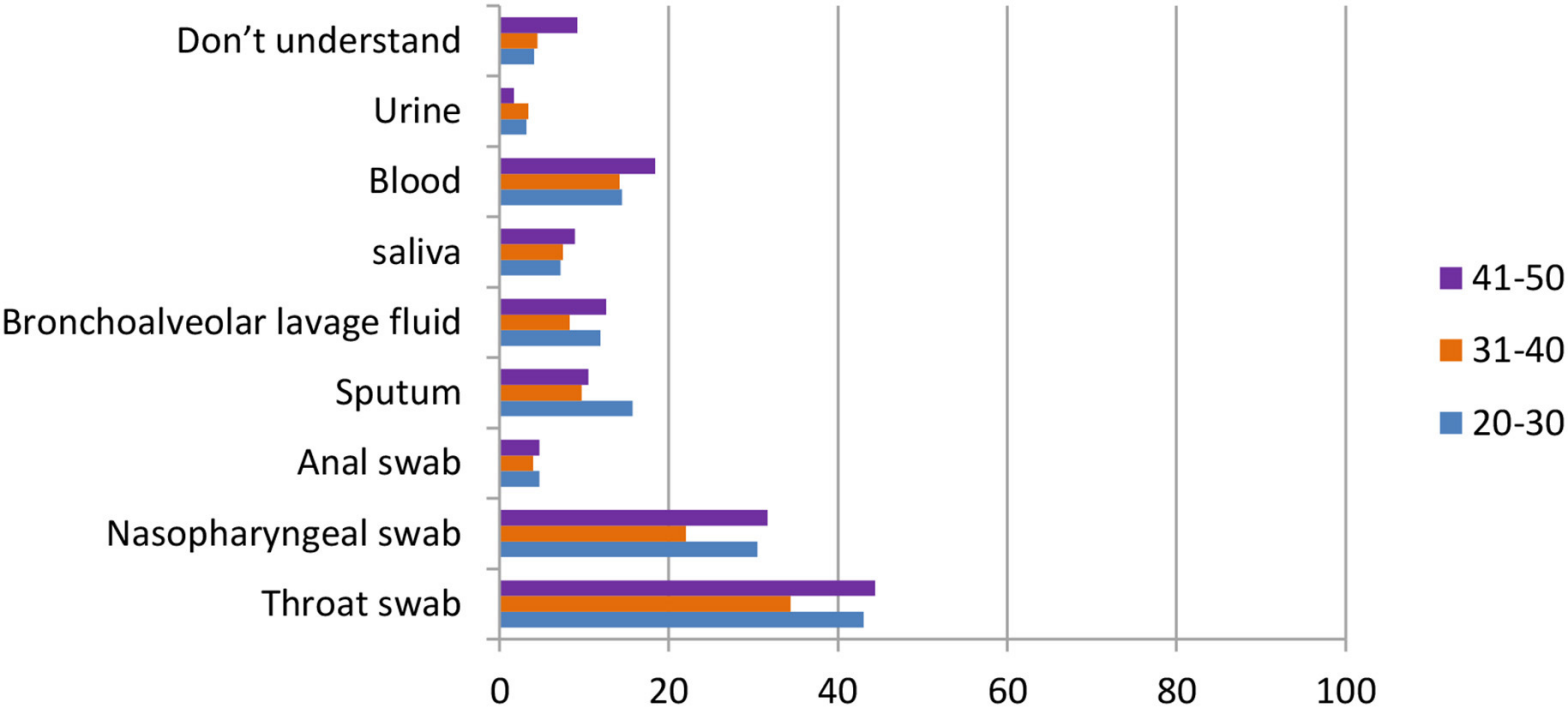
Distribution of responses to the question, “Which sampling method is the most accurate” in different age group.

In Figure 10, the data showed that in the question “which sampling method do you think is the most accurate,” both age groups 31–40 and 41–50 agreed that the throat swab was the most accurate method [128 (34.4%), 130 (44.4%)], followed by the nasopharyngeal swab [82 (22.0%), 93 (31.7%)] and the blood test [53 (14.2%), 54 (18.4%)]. On the other hand, the answer of sputum came in third [54 (15.7%)] in the age group 20–30, whereas the first two reasons were the same as the other groups [148 (43.0%), 105 (35.5%)].

### The Likert Scale of Attitude Toward the Use of the SARS-CoV-2 Testing Kit

In this section, the Likert scale was used to assess the attitude toward the use of the SARS-CoV-2 testing kit, and four questions were designed. According to the results shown in Table 4, we can observe that more people agreed it is a good thing that China has issued the emergency approval policy for novel coronavirus testing products (6.16 ± 1.30) as well as investing more in scientific research and biomedicine to improve the accuracy of detection kits (5.94 ± 1.55). Some participants also agreed that companies can sacrifice the detection time to increase detection accuracy (4.66 ± 2.00), whereas fewer participants agreed that in the development process of the detection kit, detection accuracy can be sacrificed to speed up detection (3.02 ± 2.04).

**Table 4:** The Likert scale of the attitude of the use of testing kit for SARS-CoV-2.

**Factor**	**Value**
***N***	**1,167**
Some people think that China needs to invest more in scientific research and biomedicine in order to improve the detection accuracy of testing kits. Do you agree with this opinion? mean (SD)	5.94 (1.55)
Some people think that it is a good thing that China has introduced the emergency approval policy for COVID-19 detection products. Do you agree with that? mean (SD)	6.16 (1.30)
Some people think that the detection time can be sacrificed in the development of detection kit to increase the detection accuracy. Do you agree with this opinion? mean (SD)	4.66 (2.00)
Some people think that detection accuracy can be sacrificed in the development process of detection kit, so as to accelerate the detection speed. Do you agree with this opinion? mean (SD)	3.02 (2.04)

## Discussion

The present study mainly investigated the public's knowledge and confidence in the SARS-CoV-2 detection kit. The results revealed that apart from those who were directly involved in the use of the detection kit, the public has great basic knowledge regarding the detection methods of the SARS-CoV-2 virus and the types of test kits, as well as great confidence in China in the domestic production of test kits and policy-making.

Up to now, a variety of detection methods have been available, such as RT-PCR, isothermal amplification assays, antigen, imaging, serology, neutralizing vs. binding antibodies, and so on; among them, the nucleic acid test, antigen test, and antibody test are the most recognized among the public. Results showed that the nucleic acid test remained the most common COVID-19 detection method that people have heard of, and that it is also the preferable test method. There are several kinds of nucleic acid test methods, namely PCR-based methods, regular loop-mediated isothermal amplification (LAMP)-based methods, sequence-specific LAMP-based methods, rolling circle amplification-based methods, and microarray-based methods (27). The virus nucleic acid RT-PCR test has become the current standard for the diagnosis of COVID-19. PCR is widely used for virus identification with high sensitivity and specificity (27), yet these RT-PCR test kits suffer from many limitations. For example, they have long turnaround times and are complicated in operation, averaging over 2–3 h to generate results. Besides, the PCR tests require certified laboratories, expensive equipment, and trained technicians to operate (8). There is also the risk of false-negative results. Despite the PCR, the LAMP assay is rapid and does not require expensive reagents or instruments. Therefore, the LAMP test might help reduce the cost of detecting coronavirus. Meanwhile, LAMP shows optimal performance at around 65°C, which always limits its applications and is therefore hard to operate. Rolling circle amplification has the main advantage in that it can be performed under isothermal conditions with minimal reagents and can avoid the generation of false-positive results, which is frequently encountered in PCR-based assays. The microarray assay is a detection method with rapid and high throughput. Due to its superiority, the microarray assay has been widely used in the detection of coronavirus (28).

In addition, only a few people have knowledge about antibody tests. Testing of specific antibodies of SARS-CoV-2 in patient blood is suitable for rapid, simple, highly sensitive diagnosis of COVID-19. Compared with RT-PCR, it saves time, and it does not require equipment; it is simple to perform and only requires minimal training. It will be more convenient to use fingerstick blood or heel blood instead of vein blood for out of clinic screening. However, this test cannot confirm virus presence, which only provides evidence of recent infection; it also has the risk of false-positive and false-negative results. Therefore, the combination of nucleic acid RT-PCR and the IgM–IgG antibody test can provide more accurate results (29).

The last one is the lesser-known antigen test. Coris COVID-19 Antigen Respi-Strip test (30) is a dipstick immunochromatographic test designed to detect SARS-CoV-2 antigen in nasopharyngeal secretions within 15 min. Although it has several advantages, such as the ease and fast achievement of the test, the rapid answer, the lower cost, and the non-requirement of special equipment or skills compared with molecular techniques, studies suggested that this rapid test is suffering from poor sensitivity and it is not suitable to use alone as the frontline testing for COVID-19 diagnosis (30).

Among all the testing methods, the majority of participants think that throat swab and nasopharyngeal swab are more accurate. Studies have shown that the positive rate of SARS-CoV-2 nucleic acid in sputum is higher than that of nasopharyngeal swabs. Viral nucleic acids were also detected in the blood and digestive tract (fecal/anal swabs). Simple detection of nasopharyngeal swab SARS-CoV-2 nucleic acid detection positive rate is not high, and multi-sample SARS-CoV-2 nucleic acid detection can improve the accuracy, reduce the false-negative rate, better guide clinical treatment, and evaluate the therapeutic effect (31). Saliva also plays an important role in testing; it has many benefits as a diagnostic fluid as it is easy to collect and store and contains extremely good quality DNA (32).

Although there are many detection methods for COVID-19, getting a false positive or false-negative result is still a huge issue during detection. False-negative testing of NP RT-PCR for SARS-CoV-2 is a clinically relevant problem with multiple important implications, especially in pregnant women with suspicion of severe and/or critical COVID-19 (33). There were many kinds of specimens collected from one patient, but always, only one specimen type was detected for the presence of SARS-CoV-2, which indicated that the specimen used for nucleic acid test should be collected from multiple body parts before discharge (34). Therefore, to lower the false-positive or false-negative rate, infection control measures, such as physical distancing, hand hygiene, environmental cleaning and disinfection, and adequate PPE for healthcare workers, should be strictly adhered to in order to develop and disseminate accurate diagnostic tests, assess risk levels before testing, and establish risk-stratified protocols for management of negative COVID-19 test results (34).

Nowadays, many countries produce a large number of COVID-19 test kits; a large proportion of the population, in fact, have been tested. The number of test kits should soon meet the demand. However, that alone will not solve the enormous coronavirus testing backlog (35). Having test kits will not complete the whole process of SARS-CoV-2 detection because a test is not a single device. COVID-19 testing involves several steps, each one requiring different supplies, and there are shortages at various phases of the process at different times and in different places. The healthcare labor force in some countries is not enough to meet the demand for COVID-19 virus detection, so even if these countries have enough test kits, it cannot solve the problem.

Since the COVID-19 pandemic is ongoing, there is still a continuing demand for test kits. The robust spread of the disease across the world has alarmed healthcare workers. Medical device manufacturers have increased the development and production of COVID-19 detection kits (36). Therefore, medical device manufacturers can earn a large amount of profit. The market size for COVID-19 detection kits was valued at USD 3.3 billion till now in 2020 and is expected to witness 17.3% compound annual growth rate (CAGR) from 2020 to 2026 (36). Studies showed that the oropharyngeal swab is expected to account for around USD 920 million in market value in 2020 (36). The immunoassay test strips/cassettes segment is anticipated to account for nearly USD 141 million market value in 2020, owing to the growing demand for rapid test avenues. Besides, studies showed that the diagnostic centers' segment accounted for around 32% market share in 2020 (36). These phenomena may produce problems, such as poor qualities of COVID-19 test kits and long wait times for results, and the detection process may not be vigorously pursued since manufacturers and diagnostic centers may want to earn more profit from it.

In general, most participants have basic knowledge of COVID-19 test kits. Through this study, we observed that the majority of participants have basic knowledge of COVID-19 detection, whereas healthcare workers had even higher knowledge. Since March 2020, the foreign epidemic has spread rapidly in developed countries, such as Europe and the United States. According to the WHO situation report, up to July 30, 2020, 16,812,755 cases are reported, and 662,095 death cases are recorded. Recently, the epidemic situation in third world countries, such as South America and Africa, has become more and more serious, and places, such as Hong Kong SAR, which had calmed the situation before, are now suffering a new wave of virus spread. At the same time, it has exposed the problem of an insufficient supply of COVID-19 test kits. In addition, the supply chain of ingredients for testing has been stretched to its limit, particularly for the materials used to take the virus's genetic material from the sample (37). Due to economic, labor, and production costs or other issues, the testing capacity has been delayed in some countries as well. Moreover, although there are new types of COVID-19 tests that can give more rapid results (in about 15–30 min), there is still a risk of having false-negative results. Plus, since the rapid tests use the same type of nasal swab, they would be subject to errors of sample collection, timing, and degradation (38).

## Conclusion

In this study, the survey found that the majority of participants have basic knowledge of SARS-CoV-2 virus detection methods. Most of the participants were able to identify the correct method of COVID-19 detection and the types of virus test kits. They also have great confidence in Chinese domestic production of test kits and the corresponding policy-making. All participants, including ordinary people and healthcare workers, had enough test kits and detection method information. Up until now, many countries, including the United States and Brazil, are still suffering from high rates of COVID-19. Even in China, sporadic cases still appear from time to time. Obviously, having enough knowledge about SARS-CoV-2 virus detection will benefit society during this pandemic. However, easing anxiety about the pandemic does not depend only on great knowledge of virus detection methods, and whether high compliance rates and knowledge of SARS-CoV-2 virus detection methods contribute to the pandemic problem remains unknown.

## Data Availability Statement

The original contributions presented in the study are included in the article/supplementary materials, further inquiries can be directed to the corresponding author/s.

## Ethics Statement

Ethical review and approval was not required for the study on human participants in accordance with the local legislation and institutional requirements. The patients/participants provided their written informed consent to participate in this study.

## Author Contributions

RL, KL, and ZH designed the online questionnaire and collected the data. RS was in charge of the manuscript. CZ and W-KM reviewed the manuscript and provide additional support. All authors contributed to the article and approved the submitted version.

## Conflict of Interest

The authors declare that the research was conducted in the absence of any commercial or financial relationships that could be construed as a potential conflict of interest.

## References

[1] Who.int. (2020) Available online at: https://www.who.int/docs/default-source/coronaviruse/situation-reports/20200730-covid-19-sitrep-192.pdf?sfvrsn=5e52901f_8 (accessed August 8, 2020).

[2] Sanders J Monogue M Jodlowski T Cutrell J. Pharmacologic treatments for coronavirus disease 2019 (COVID-19). JAMA. (2020) 323:1824–36. 10.1001/jama.2020.6019 32282022

[3] Khan K Dimtri F Vargas C Surani S. COVID-19: a review of emerging preventative vaccines and treatment strategies. Cureus. (2020) 12:e8206. 10.7759/cureus.8206 32577324 PMC7305574

[4] Chiappelli F. Comments on “An insertion unique to SARS-CoV-2 exhibits super antigenic character strengthened by recent mutations” by Cheng MH et al. 2020. Bioinformation. (2020) 16:474–6. 10.6026/97320630016474 32884212 PMC7452748

[5] Qian M Jiang J COVID-19 and social distancing. J Public Health. (2020). [Epub ahead of print]. 10.1007/s10389-020-01321-z. PMC7247774 32837835

[6] Zhang X Zhou H Zhang W Dou Q Li Y Wei J. Assessment of coronavirus disease 2019 community containment strategies in Shenzhen, China. JAMA Netwk Open. (2020) 3:e2012934. 10.1001/jamanetworkopen.2020.12934 32568401 PMC7309437

[7] Coronavirus disease 2019 (COVID-19) - Diagnosis and Treatment - Mayo Clinic (2020). Available online at: https://www.mayoclinic.org/diseases-conditions/coronavirus/diagnosis-treatment/drc-20479976 (accessed August 19, 2020).

[8] Li Z Yi Y Luo X Xiong N Liu Y Li S. Development and clinical application of a rapid IgM-IgG combined antibody test for SARS-CoV-2 infection diagnosis. J Med Virol. (2020) 92:1518–24. 10.1002/jmv.25727 32104917 PMC7228300

[9] La Marca A Capuzzo M Paglia T Roli L Trenti T Nelson S. Testing for SARS-CoV-2 (COVID-19): a systematic review and clinical guide to molecular and serological in-vitro diagnostic assays. Reprod Biomed Online. (2020) 41:483–99. 10.1016/j.rbmo.2020.06.001 32651106 PMC7293848

[10] Vinh D Zhao X Kiong K Guo T Jozaghi Y Yao C. Overview of COVID−19 testing and implications for otolaryngologists. Head Neck. (2020) 42:1629–33. 10.1002/hed.26213 32342570 PMC7267427

[11] Yip C Ho C Chan J To K Chan H Wong S. Development of a novel, genome subtraction-derived, SARS-CoV-2-specific COVID-19-nsp2 real-time RT-PCR assay and its evaluation using clinical specimens. Int J Mol Sci. (2020) 21:2574. 10.3390/ijms21072574 32276333 PMC7177594

[12] Xiao A Tong Y Zhang S. False negative of RT-PCR and prolonged nucleic acid conversion in COVID-19: rather than recurrence. J Med Virol. (2020) 92:1755–56. 10.1002/jmv.25855 32270882 PMC7262304

[13] Long D Gombar S Hogan C Greninger A O'Reilly-Shah V Bryson-Cahn C Occurrence and timing of subsequent severe acute respiratory syndrome coronavirus 2 reverse-transcription polymerase chain reaction positivity among initially negative patients. Clin Infect Dis. (2020). [Epub ahead of print]. 10.1093/cid/ciaa722. PMC7314163 33501950

[14] Wang P. Combination of serological total antibody and RT-PCR test for detection of SARS-COV-2 infections. J Virol Methods. (2020) 283:113919. 10.1016/j.jviromet.2020.113919 32554043 PMC7295513

[15] Zhong B Luo W Li H Zhang Q Liu X Li W. Knowledge, attitudes, and practices towards COVID-19 among Chinese residents during the rapid rise period of the COVID-19 outbreak: a quick online cross-sectional survey. Int J Biol Sci. (2020) 16:1745–52. 10.7150/ijbs.45221 32226294 PMC7098034

[16] Zhang M Zhou M Tang F Wang Y Nie H Zhang L. Knowledge, attitude, and practice regarding COVID-19 among healthcare workers in Henan, China. J Hosp Infect. (2020) 105:183–7. 10.1016/j.jhin.2020.04.012 32278701 PMC7194961

[17] Lin Y Hu Z Alias H Wong L Knowledge, attitudes, impact, and anxiety regarding COVID-19 infection among the public in China. Front Public Health. (2020) 8:236 10.3389/fpubh.2020.00236 32574305 PMC7266871

[18] Chan E Huang Z Lo E Hung K Wong E Wong S. Sociodemographic predictors of health risk perception, attitude and behavior practices associated with health-emergency disaster risk management for biological hazards: the case of COVID-19 pandemic in Hong Kong, SAR China. Int J Environ Res Public Health. (2020) 17:3869. 10.3390/ijerph17113869 32485979 PMC7312582

[19] Clements J. Knowledge and behaviors toward COVID-19 among US residents during the early days of the pandemic: cross-sectional online questionnaire. JMIR Public Health Surveill. (2020) 6:e19161. 10.2196/19161 32369759 PMC7212816

[20] Alzoubi H Alnawaiseh N Al-Mnayyis A Abu-Lubad M Aqel A Al-Shagahin H COVID-19 - knowledge, attitude and practice among medical and non-medical university students in Jordan. J Pure Appl Microbiol. (2020) 14:17–24. 10.22207/JPAM.14.1.04

[21] Honarvar B Lankarani K Kharmandar A Shaygani F Zahedroozgar M Rahmanian Haghighi M. Knowledge, attitudes, risk perceptions, and practices of adults toward COVID-19: a population and field-based study from Iran. Int J Public Health. (2020) 65:731–9. 10.1007/s00038-020-01406-2 32583009 PMC7311321

[22] Parikh P Shah B Phatak A Vadnerkar A Uttekar S Thacker N. COVID-19 Pandemic: knowledge and perceptions of the public and healthcare professionals. Cureus. (2020) 12:e8144. 10.7759/cureus.8144 32550063 PMC7294885

[23] Al-Hanawi M Angawi K Alshareef N Qattan A Helmy H Abudawood Y. Knowledge, attitude and practice toward COVID-19 among the public in the Kingdom of Saudi Arabia: a cross-sectional study. Front Public Health. (2020) 8:217. 10.3389/fpubh.2020.00217 32574300 PMC7266869

[24] Riccò M Ferraro P Gualerzi G Ranzieri S Henry B Said Y. Point-of-care diagnostic tests for detecting SARS-CoV-2 antibodies: a systematic review and meta-analysis of real-world data. J Clin Med. (2020) 9:1515. 10.3390/jcm9051515 32443459 PMC7290955

[25] Kebede Y Yitayih Y Birhanu Z Mekonen S Ambelu A. Knowledge, perceptions and preventive practices towards COVID-19 early in the outbreak among Jimma university medical center visitors, Southwest Ethiopia. PLoS ONE. (2020) 15:e0233744. 10.1371/journal.pone.0233744 32437432 PMC7241810

[26] Kumar J Katto M Siddiqui A Sahito B Jamil M Rasheed N. Knowledge, attitude, and practices of healthcare workers regarding the use of face mask to limit the spread of the new coronavirus disease (COVID-19). Cureus. (2020) 12:e7737. 10.7759/cureus.7737 32455057 PMC7241223

[27] Wu J Liu J Li S Peng Z Xiao Z Wang X. Detection and analysis of nucleic acid in various biological samples of COVID-19 patients. Travel Med Infect Dis. (2020) 37:101673. 10.1016/j.tmaid.2020.101673 32311437 PMC7165102

[28] Shen M Zhou Y Ye J Abdullah AL-maskri A Kang Y Zeng S. Recent advances and perspectives of nucleic acid detection for coronavirus. J Pharm Anal. (2020) 10:97–101. 10.1016/j.jpha.2020.02.010 32292623 PMC7102540

[29] Xiang F Wang X He X Peng Z Yang B Zhang J. Antibody detection and dynamic characteristics in patients with coronavirus disease 2019. Clin Infect Dis. (2020). 10.1093/cid/ciaa461 32306047 PMC7188146

[30] Scohy A Anantharajah A Bodéus M Kabamba-Mukadi B Verroken A Rodriguez-Villalobos H. Low performance of rapid antigen detection test as frontline testing for COVID-19 diagnosis. J Clin Virol. (2020) 129:104455. 10.1016/j.jcv.2020.104455 32485618 PMC7240272

[31] Xie C Lu J Wu D Zhang L Zhao H Rao B. False negative rate of COVID-19 is eliminated by using nasal swab test. Travel Med Infect Dis. (2020) 37:101668. 10.1016/j.tmaid.2020.101668 32283215 PMC7151360

[32] Sri Santosh T Parmar R Anand H Srikanth K Saritha M. A review of salivary diagnostics and its potential implication in detection of Covid-19. Cureus. (2020) 12:e7708. 10.7759/cureus.7708 32313785 PMC7164701

[33] West C Montori V Sampathkumar P. COVID-19 testing. Mayo Clin Proc. (2020) 95:1127–9. 10.1016/j.mayocp.2020.04.004 32376102 PMC7151274

[34] Renauer C Thermo Fisher Scientific to Produce Millions of COVID-19 Test Kits in the Next Few Weeks. The Motley Fool Available online at: https://www.fool.com/investing/2020/03/16/thermo-fisher-millions-covid-19-test-kits-soon.aspx. Published 2020 (accessed August 8, 2020).

[35] COVID-19 Detection Kits Market Size & Share. Industry Report 2020-2026. Global Market Insights, Inc (2020). Available online at: https://www.gminsights.com/industry-analysis/covid-19-detection-kits-market (accessed August 8, 2020).

[36] Coronavirus Testing Shortages: What's the Problem? (2020). Available online at: https://www.ft.com/content/86efe246-692e-11ea-800d-da70cff6e4d3 (accessed August 8, 2020).

[37] The Problems With COVID-19 Testing. (And it's Not What You Think). (2020). Available online at: https://www.al.com/opinion/2020/04/the-problems-with-covid-19-testing-and-its-not-what-you-think.html (accessed August 8, 2020).

[38] Mak G Cheng P Lau S Wong K Lau C Lam E. Evaluation of rapid antigen test for detection of SARS-CoV-2 virus. J Clin Virol. (2020) 129:104500. 10.1016/j.jcv.2020.104500 32585619 PMC7278630

